# Primary oral leiomyosarcoma of the maxillary bone and sinus: case report and up-to-date review of literature

**DOI:** 10.1016/j.bjorl.2021.07.013

**Published:** 2021-10-17

**Authors:** Niccolò Lombardi, Elena M. Varoni, Dimitri Rabbiosi, Marco Cucurullo, Laura Moneghini, Giovanni Lodi

**Affiliations:** a Università degli Studi di Milano, Dipartimento di Scienze Biomediche Chirurgiche e Odontoiatriche, Milan, Italy; b ASST Santi Paolo e Carlo, Odontostomatology II Unit, Milan, Italy; c ASST Santi Paolo e Carlo, Maxillofacial Surgery Unit, Milan, Italy; d ASST Santi Paolo e Carlo, Human Pathology Unit, Milan, Italy

## Introduction

Leiomyosarcoma (LMS) is a mesenchymal malignancy of smooth muscle, which accounts for 1%–4% of head and neck sarcomas.^1^, ^2^ The involvement of the oral cavity is considered extremely rare.^1^ Due to the lack of smooth muscle tissue in the oral cavity, it has been suggested that LMS arise from smooth muscle cells of the blood vessel walls, circumvallate papillae of the tongue, neuromuscular bundles, myoepithelial cells or pluripotent undifferentiated mesenchymal cells.^1^, ^3^, ^4^

Clinically, sarcomas of the oral cavity show nonspecific signs and symptoms and can appear as primary tumors, radiation-associated tumors or metastatic tumors.^1^, ^4^, ^5^ Biopsy, histological examination and immunohistochemical staining are mandatory to achieve a definitive diagnosis.^1^, ^5^

The aim of this study was to report a case of maxillary LMS and to perform a review of literature including papers describing LMS of the oral cavity.

## Methods

An electronic search in PubMed (National Library of Medicine) was performed including the previous reported cases of primary oral leiomyosarcoma from the year 2000 to March 2021. The terms “oral leiomyosarcoma” (295 results), “leiomyosarcoma tongue” (28 results), “leiomyosarcoma maxilla” (23 results), “leiomyosarcoma mandible” (31 results) and “leiomyosarcoma buccal” (13 results) were used. Cases were considered as “oral” if they primarily arose in mandible, maxillary bone, gingiva, buccal mucosa, lips, palate, tongue, and floor of the mouth. Only the article in English language were included in the final analysis. We excluded the articles which did not report complete immunohistochemical data related to LMS diagnosis. We included only the cases of primary oral LMS which stated, within the abstract or the manuscript, the immunohistochemical positivity for Smooth Muscle Actin (SMA) or at least for two of these markers: muscle specific actin (HHF35), desmin, h-caldesmon.^6^ When the full-text was not available, we directly contacted the authors.

## Case report and literature review

A 67-year-old man presented at the oral medicine department for oral growing-mass, which caused difficult in feeding and speaking. The patient reported the first occurrence of a small swelling on the premaxilla’s palatal side approximately three months before, but he did not seek medical care due to the COVID-19 pandemic lockdown. He reported sporadic bleeding episodes and progressive increasing of lesion’s dimensions up to our observation. The clinical history was negative for systemic diseases and drug intake.

**Figure 1:**
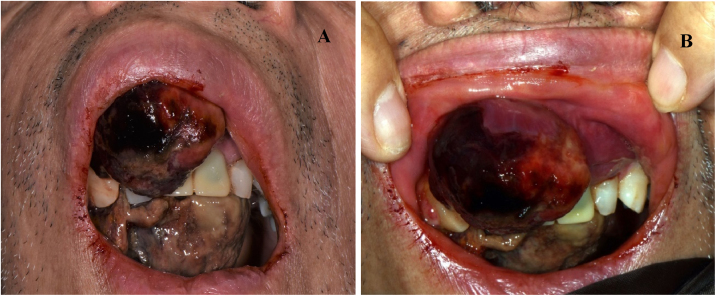
Clinical picture of a large growing mass of the upper maxilla extended on both buccal and palatal sides (A; B).

**Figure 2:**
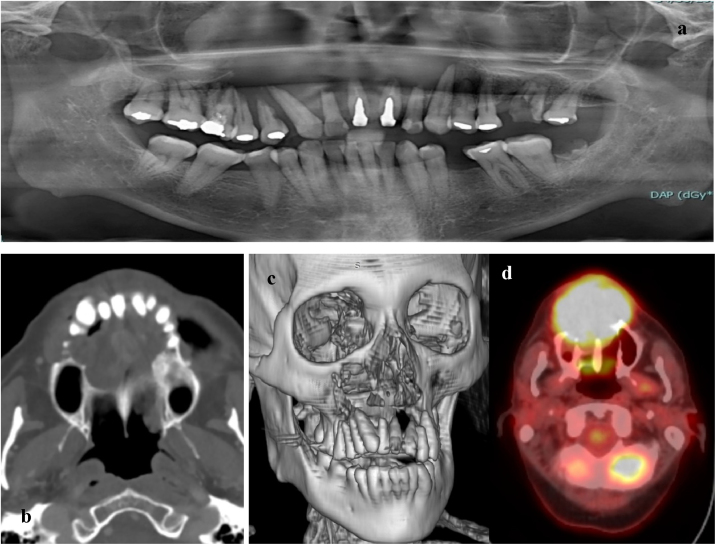
Wide maxillary osteolytic lesion associated with dental root resorption and involvement of the right maxillary sinus: orthopantomography (a), axial CT (b), 3D reconstruction (c) and PET (d) images.

**Figure 3:**
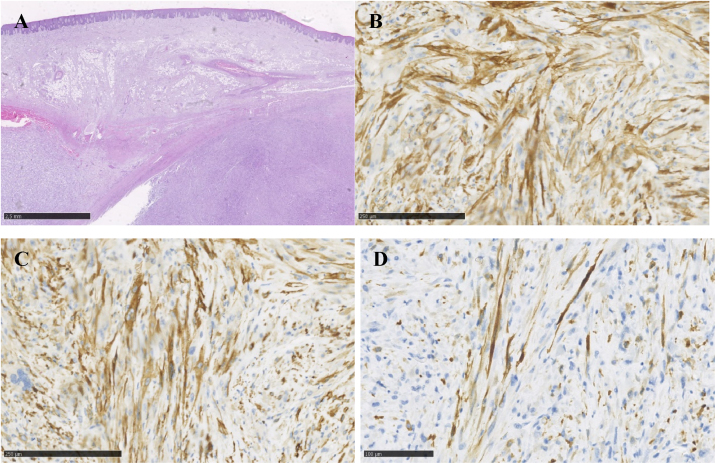
Hematoxylin and eosin stains show, under the oral epithelium, a sarcomatous proliferation characterized by fascicles of medium/large size spindle cells with abundant eosinophilic cytoplasm and well-defined cell borders (A). The nuclei are spindle or oval with mild to marked atypia. Both typical and atypical mitotic activity is easily found together a mild lymphocytic infiltrate without necrosis. Immunochemistry shows diffuse expression of smooth muscle actin (B) and HHF35 (C), and desmin (D) at a minor degree.

**Figure 4:**
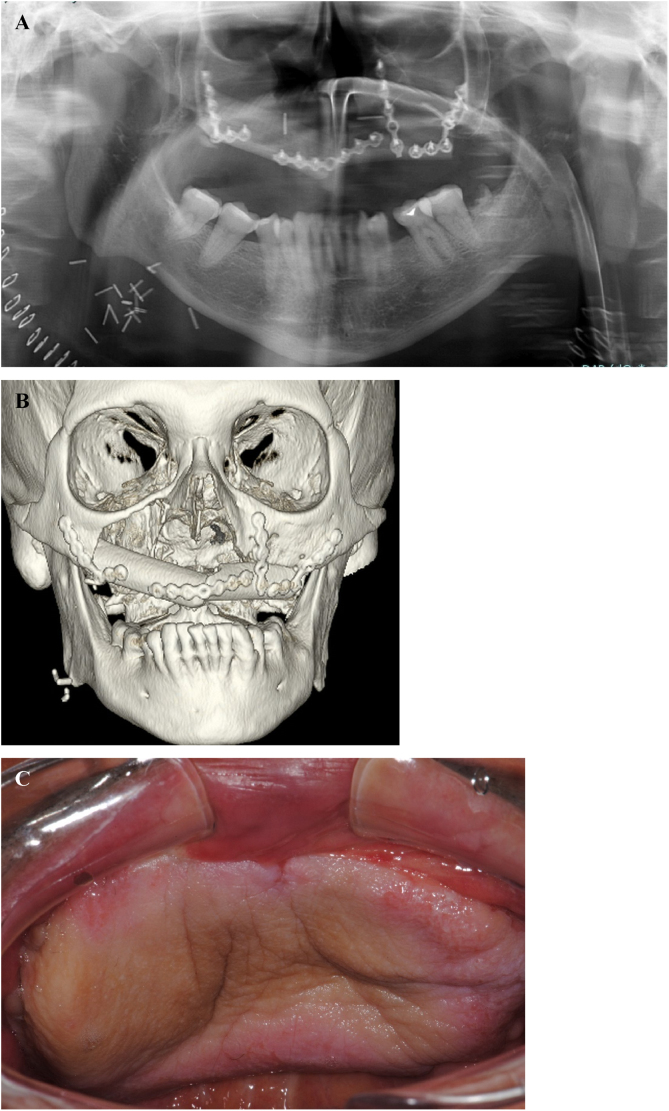
Total maxillectomy and reconstruction with fibula free flap: orthopantomography (A) and 3D-CT images (B); clinical picture at follow-up examination, 6-months after surgical treatment (C).

At the clinical examination, the patient showed impairment in speaking, due to a large painless brownish-reddish mass, which appeared bilobular and firm at palpation. The lesion had a total longitudinal size of 6 cm, it was extended at the buccal side of the premaxilla, covering the hard palate, and incorporating the teeth (Fig. 1). Orthopantomography and CT revealed a wide area of osteolysis of the maxillary and palatal bones, which bilaterally involved the nasal fossa and the right maxillary sinus and caused the dental root resorption (Fig. 2). Putative clinical diagnosis included mesenchymal tumors, Kaposi’s Sarcoma, hematologic or bone malignancies.^7^, ^8^, ^9^ Multiple incisional biopsies led to the histopathological diagnosis of high-grade leiomyosarcoma. Immunohistochemical profile of the lesion was positive for smooth-muscle-actin and muscle-specific antigen (HHF-35), shows minor-degree expression for desmin and was negative for S-100 protein (Fig. 3). Positron emission tomography showed large enhancing maxillary lesion (SUV_max_ 15.8), and few areas, putatively inflammatory, localized at the esophagus and mediastinum (SUV_max_ 2.2) (Fig. 2). Because of the patient’s good systemic condition (ECOG Performance Status scale grade 1), two cycles of neo-adjuvant chemotherapy with ifosfamide, doxorubicin and mesna were performed, before surgery, with subsequent reduction of tumour’s size. Total maxillectomy (with free resection margins) followed by cervical lymphadenectomy and reconstruction with fibula-free flap was performed (Fig. 4). After surgical resection, the patient received three further cycles of doxorubicin and dacarbazine, as adjuvant chemotherapy. The patient is currently on follow-up and, eight-months after surgery, he does not show clinical or radiological signs of recurrence (Fig. 4).

Table 1 listed the cases found according to this literature review. A total of 73 cases of oral primary LMS have been identified, 42 (57.5%) in women and 31 (42.5%) in men, with a median age of 48 years old. Oral LMS arose in 35 (48%) cases from the soft tissues and in 38 (52%) cases from the hard tissues (Table 2).

**Table 1:** Reported cases of oral primary leiomyosarcomas in the last two decades, excluding three manuscripts for which the full text was not available and the immunohistochemical details were not provided in the abstract (modified from).^3^

Author(s)	Year of publication	Patient gender	Patient age (years)	Primary location
Lombardi et al. (present case)	2021	M	67	Hard tissue, maxilla
Correia Neto IJ et al.^16^	2021	F	72	Soft tissue, buccal mucosa
Choi YS et al.^11^	2020	M	62	Hard tissue, maxilla
		F	61	Hard tissue, mandible
		M	66	Hard tissue, maxilla
		F	48	Hard tissue, mandible
		F	36	Hard tissue, maxilla
		F	23	Hard tissue, mandible, and maxilla
		M	16	Hard tissue, mandible
		F	55	Hard tissue, mandible
Bala M et al.^17^	2019	M	54	Hard tissue, mandible
Agarwal M et al.^18^	2018	F	45	Soft tissue, tongue
Sumida T et al.^19^	2018	M	29	Hard tissue, mandible
Ko ME et al.^5^	2018	M	86	Soft tissue, buccal mucosa
Viviano M et al.^20^	2017	F	22	Soft tissue, mandible
Li RH et al.^21^	2017	F	54	Soft tissue, floor of the mouth
Kenea TT et al.^22^	2017	M	12	Soft tissue, mandible
Lewandowski B et al.^23^	2016	F	58	Hard tissue, mandible
Suarez-Alen F et al.^1^	2014	F	66	Soft tissue, buccal mucosa
Sandhu SV et al.^24^	2014	M	63	Hard tissue, maxilla
Moghadam SA et al.^25^	2014	F	67	Hard tissue, mandible
Nagpal DKJ et al.^26^	2013	F	35	Soft tissue, buccal mucosa
Patel K et al.^27^	2013	M	23	Hard tissue, mandible
Schutz A et al.^28^^a^	2013	M	75	Soft tissue, lip
		F	39	Hard tissue, mandible
		M	25	Soft tissue, floor of the mouth
Rege IC et al.^29^	2013	M	64	Hard tissue, mandible
Taghipour Zahir S et al.^30^	2013	M	24	Hard tissue, maxilla
Divyambika CV et al.^31^	2012	F	8	Soft tissue, buccal mucosa
Ahn JH et al.^32^	2012	F	54	Soft tissue, tongue
Riaz N et al.^33^	2011	M	65	Hard tissue, maxilla
Chew IK et al.^34^	2009	M	36	Hard tissue, maxilla
Santana MVM et al.^35^	2008	M	14	Soft tissue, hard palate
Mendonça EF et al.^36^	2008	F	9	Soft tissue, gingiva
Yadav R et al.^37^	2008	F	27	Soft tissue, buccal mucosa
Crossman T et al.^10^	2008	F	46	Soft tissue, tongue
Pinheiro J et al.^38^	2007	F	40	Hard tissue, mandible
Ethunandan M et al.^14^	2007	F	79	Soft tissue, tongue
		F	97	Soft tissue, tongue
		F	50	Hard tissue, maxilla
		M	51	Soft tissue, buccal mucosa
Rodini CO et al.^39^	2007	F	54	Soft tissue, mandible
		M	63	Hard tissue, maxilla
Castaldi A ^40^	2006	F	52	Soft tissue, tongue
Yang S‐W et al.^41^	2006	F	54	Soft tissue, tongue
Vilos GA et al.^12^	2005	F	26	Hard tissue, mandible
		M	48	Hard tissue, mandible
		F	30	Soft tissue, soft palate
		F	20	Hard tissue, maxilla
Cheng C‐Y et al.^42^	2004	F	9	Soft tissue, mandible
Prasad KC et al.^43^	2004	F	27	Hard tissue, maxilla
		F	42	Hard tissue, maxilla
Ikram M et al.^44^	2003	M	41	Hard tissue, maxilla
Lo Muzio L et al.^45^	2002	F	31	Soft tissue, maxilla
Montgomery E et al.^46^^b^	2002	M	37	Hard tissue, maxilla
		F	21	Soft tissue, tongue
		F	34	Hard tissue, maxilla
		M	42	Soft tissue, buccal mucosa
Wada S et al.^47^	2002	F	71	Hard tissue, maxilla
Nikitakis NG et al.^48^	2002	M	35	Soft tissue, mandible
		F	51	Hard tissue, mandible
Dios PD et al.^49^	2001	M	67	Soft tissue, soft palate
Sumida T et al.^50^	2001	M	77	Hard tissue, maxilla
Dry et al.^51^^c^	2000	M	31	Hard tissue, maxilla
		M	58	Hard tissue, maxilla
		F	88	Soft tissue, floor of the mouth
		M	28	Hard tissue, maxilla
		F	74	Hard tissue, mandible
		F	34	Soft tissue, palate
		F	91	Soft tissue, lip
		F	27	Soft tissue, mandible
		M	N/A	Soft tissue, gingiva
Lo Muzio L et al.^52^	2000	M	67	Soft tissue, tongue

a Case nº 4 was excluded (SMA, HHF35 and desmin unknown).

b Cases nº 8, 11, 12 excluded (positive only for desmin; SMA and HHF35 unknown).

c Case nº 6 was excluded (positive only for desmin; SMA and HHF35 not performed).

**Table 2:** Distribution of the LMS reported cases considering the arising tissues.

Hard tissues (n = 38)		Soft tissues (n = 35)	
Maxillary bone	21	Tongue	9
Mandible	15	Buccal Mucosa	8
Maxilla and mandible	1	Mandibular soft tissues	6
		Palate (hard and soft)	4
		Mouth-floor	3
		Gingiva	2
		Lip	2
		Maxillary soft tissues	1

## Discussion

Leiomyosarcoma is a malignant tumor of smooth muscle derivation which account for 5%–10% of all soft tissue sarcomas.^3^, ^10^ LMS frequently affects the retro-peritoneal region, the uterus and the gastrointestinal tract.^1^, ^11^ Oral LMS are extremely rare, accounting for 0.64% of all LMS and for 5.7% of head and neck LMS.^5^, ^11^ The jawbones appear the most frequently affected oral sites (70% of cases) followed by tongue, buccal mucosa, soft palate, upper lip and floor of the mouth,^1^, ^5^, ^11^, ^12^ although a recent systematic review, which reported published case of intra-oral LMS up to 2017, found that soft tissues were more frequently involved than bone.^3^ Oral LMS can occur at any age, commonly in the 5–7^th^ decades, with similar gender incidence rate (M:F, 11:9).^3^, ^11^ Clinically, it appears as a growing mass, painless and firm, which infiltrate the surrounding tissues with no pathognomonic signs.^1^, ^3^

Due to its rarity, oral LMS can be misdiagnosed with other common benign lesions before biopsy.^11^ Moreover, due to overlapping histological features between different types of head and neck sarcomas, this lesion could be also misclassified for other more common spindle cell tumors of the oral cavity.^3^, ^5^, ^13^ For this reason, histological examination associated with immunohistochemical confirmation is mandatory to obtain a definitive diagnosis of oral LMS.^1^, ^3^, ^11^, ^13^

Surgical resection with tumour-free margins is widely accepted as the treatment of choice for oral LMS.^1^, ^3^, ^5^, ^11^ In cases where the anatomical location of tumor does not allow radical surgical intervention, radiotherapy and administration of neoadjuvant/adjuvant chemotherapy may improve survival time and decrease or delay the recurrence rate.^1^, ^2^, ^11^ However, no standard of care is still available.^2^

The prognosis for oral LMS is poor, associated with a high rate of recurrence (42% for high-grade LMS within 2 years from surgery) and regional or distant metastasis.^1^, ^2^, ^5^ Unfavorable clinical course is related to several factors, such as delayed diagnosis, tumor size (larger than 4 cm), histopathological high-grade (poorly-differentiated), site (bone involvement and extension into paranasal sinuses), lymphatic or hematogenous dissemination, positive surgical margins.^1^, ^2^, ^3^, ^5^ The estimated 5-years survival rate for primary oral LMS range from 55% to 61%.^1^, ^12^, ^14^ Periodic follow-up examinations are mandatory for an early diagnosis of any signs of recurrence.^1^, ^2^, ^11^

In the case presented above, the delayed diagnosis is associated with an increased size of the tumor and a subsequent more aggressive surgical intervention; it is not possible to establish how much the delayed diagnosis could have influenced the prognosis for the patient. However, on our experience during the first months of COVID pandemic, patients were often more scared of contracting COVID-infection in the hospital rather than their neoplastic disease.^15^

## Conclusion

Even if extremely rare, LMS should be considered in the differential diagnosis of oral growing-mass. Early diagnosis and treatment are essential to reduce the risk of recurrence and guarantee better prognosis. Periodic follow-up examinations are mandatory to identify early any signs of recurrence.^1^, ^2^, ^11^ Due to the rarity of oral LMS further studies are necessary to better characterize this disease.

## Institution’s ethics committee

Institution’s ethics committee approval is not required for the case report.

The study was conducted in compliance with the recognized international standards, including the principles of the Declaration of Helsinki.

## Patient’s informed consent

Data and samples were collected under patient’s informed written consent, guaranteeing anonymity.

## Funding

The authors received no specific funding for this work. No funds, grants, or other support were received.

## Conflicts of interest

The authors declare no conflicts of interest.

## References

[1] Suárez-Alén F., Otero-Rey E., Peñamaría-Mallõn M., García-García A., Blanco-Carriõn A. (2015). Oral leiomyosarcoma: the importance of early diagnosis. Gerodontology.

[2] Saluja T.S., Iyer J., Singh S.K. (2019). Leiomyosarcoma: prognostic outline of a rare head and neck malignancy. Oral Oncol.

[3] Ko E. (2019). Primary oral leiomyosarcoma: A systematic review and update. J Oral Pathol Med.

[4] Azevedo R.S., Pires F.R., Gouvêa A.F., Lopes M.A., Jorge J. (2012). Leiomyosarcomas of the oral cavity: Report of a radiation-associated and a metastatic case. Oral Maxillofac Surg.

[5] Ko E.M., McHugh J.B. (2018). Primary leiomyosarcoma of the buccal mucosa: report of a case and review of the literature. Head Neck Pathol.

[6] El-Naggar A.K., Chan J.K.C., Grandis J.R., Takata T., Slootweg P. (2017).

[7] Lombardi N., Varoni E., Sardella A., Lodi G. (2020). Oral Kaposi’s sarcoma in a HIV-negative young patient. Oral Oncol.

[8] Lombardi N., Flora A., Franchini R., Sorrentino D., Lodi G., Varoni E.M. (2019). Gingival localisation of extramedullary multiple myeloma. Lancet Oncol.

[9] Lombardi N., Varoni E.M., Sorrentino D., Nicali A., Lodi G. (2022). Growing gingival mass in a patient affected by hyperparathyroidism. J Am Dent Assoc.

[10] Crossman T., Ward P., Herold J. (2008). Leiomyosarcoma of the tongue: a case report. Br J Oral Maxillofac Surg.

[11] Choi Y.S., Almansoori A.A., Jung T.Y., Lee J.I., Kim S.M., Lee J.H. (2020). Leiomyosarcoma of the jaw: Case series. J Korean Assoc Oral Maxillofac Surg.

[12] Vilos G.A., Rapidis A.D., Lagogiannis G.D., Apostolidis C. (2005). Leiomyosarcomas of the oral tissues: Clinicopathologic analysis of 50 cases. J Oral Maxillofac Surg.

[13] Lombardi N., Varoni Em, Bazzacchi R., Moneghini L., Lodi G. (2021). Secondary undifferentiated pleomorphic sarcoma of the mandible in a HIV patient who underwent radiotherapy for oral carcinoma. Spec Care Dent.

[14] Ethunandan M., Stokes C., Higgins B., Spedding A., Way C., Brennan P. (2007). Primary oral leiomyosarcoma: a clinico-pathologic study and analysis of prognostic factors. Int J Oral Maxillofac Surg.

[15] Sardella A., Varoni E., Carrassi A., Pispero A., Lombardi N., Lodi G. (2021). Who's afraid of the big bad wolf? The experience of an Oral Medicine Unit in the time of Corona-Virus. Oral Dis..

[16] Correia Neto I.J., Cunha J.L.S., de Oliveira C.E., de Almeida O.P., Aciole dos Santos G.T., Freitas M.M.D. (2021). A recurrent leiomyosarcoma of the buccal mucosa: An immunohistochemistry study and literature review. Oral Oncol..

[17] Bala M., Ray A., Saraf A. (2019). Leiomyosarcoma of Mandible: A Diagnostic Dilemma; Case Report and Review of Literature. Indian J Otolaryngol Head Neck Surg.

[18] Agarwal M., Agarwal L., Mathur V., Khandelwal G. (2018). A rare case report of leiomyosarcoma of tongue. AME Case Reports.

[19] Sumida T., Ishikawa A., Nakano H., Kobayashi Y., Yamada T., Mori Y. (2018). Mandibular central leiomyosarcoma with high telomerase activity: a case report. Int J Clin Exp Pathol.

[20] Viviano M., Miracco C., Lorenzini G., Baldino G., Cocca S. (2018). Gingival Leiomyosarcoma in a Young Woman: Case report and literature review. Sultan Qaboos Univ Med J [SQUMJ].

[21] Li R.-H., Liu S.-H. (2017). Primary leiomyosarcoma in the floor of mouth: a case report. Int J Clin Exp Pathol.

[22] Kenea T., Kebede B., Gozjuze F., Kiros H., Wilde F. (2017). Primary leiomyosarcoma of the mandibular alveolar mucosa of a 12-year-old child from ethiopia: a case report. Craniomaxillofac Trauma Reconstr.

[23] Lewandowski B., Brodowski R., Pakla P., Stopyra W., Gawron I. (2016). Leiomyosarcoma in the mandible. Medicine (Baltimore).

[24] Sandhu S., Sodhi S.S., Rai S., Bansal H. (2014). Primary leiomyosarcoma of the maxilla: An investigative loom-report of a challenging case and review of literature. J Oral Maxillofac Pathol.

[25] Mokhtari S., Moghadam S., Khodayari A. (2014). Primary leiomyosarcoma of the mandible. J Oral Maxillofac Pathol.

[26] Nagpal D.K., Prabhu P., Shah A., Palaskar S. (2013). Leimyosarcoma of the buccal mucosa and review of literature. J Oral Maxillofac Pathol.

[27] Patel K., French C., Khariwala S.S., Rohrer M., Kademani D. (2013). Intraosseous leiomyosarcoma of the mandible: A case report. J Oral Maxillofac Surg.

[28] Schütz A., Smeets R., Driemel O., Hakim S.G., Kosmehl H., Hanken H. (2013). Primary and Secondary Leiomyosarcoma of the Oral and Perioral Region-Clinicopathological and Immunohistochemical Analysis of a Rare Entity With a Review of the Literature. J Oral Maxillofac Surg.

[29] Rege I.C.C., Costa N.L., Batista A.C., da Silva C.M., Meneghini A.J., Mendonça E.F. (2013). High-grade primary leiomyosarcoma in the mandible: Diagnosis and treatment. Head Neck.

[30] Taghipour Zahir S., Sharahjin N.S. (2013). Leiomyosarcoma of the maxilla in a 24-year-old man who initially presented with odontalgia, and suffered from tumour mismanagement. Case Reports.

[31] Divyambika C.V., Sathasivasubramanian S., Krithika C.L., Malathi N., Prathiba D. (2012). Pediatric oral leiomyosarcoma: Rare case report. J Cancer Res Ther.

[32] Ahn J.H., Mirza T., Ameerally P. (2012). Leiomyosarcoma of the tongue with multiple metastases: a case report and review of literature. J Oral Maxillofac Surg.

[33] Riaz N., Warriach R.A., Aftab A. (2011). Massive leiomyosarcoma of the maxilla. J Ayub Med Coll Abbottabad.

[34] Chew Y.K., Noorizan Y., Khir A., Brito-Mutunayagam S. (2009). Leiomyosarcoma of the maxillary sinus. Med J Malaysia.

[35] Santana M.V.M., Duarte E.C.B., Johann A.C.B.R., de Fátima Correia-Silva J., de Aguiar M.C.F., Mesquita R.A. (2008). Ulcerated midline nodule of the hard palate. Oral Surgery, Oral Med Oral Pathol Oral Radiol Endodontology.

[36] Mendonça E.F., Martins Da Silva C., Meneghini A.J., Silva G.B.L., Filho J.A.A., Batista A.C. (2008). Low-grade gingival leiomyosarcoma in a child. J Dent Child.

[37] Yadav R., Bharathan S. (2008). Leiomyosarcoma of the buccal mucosa: a case report with immunohistochemistry findings. J Oral Sci.

[38] Pinheiro Jde J., Alves Sde M., Okuda E., Jorge W.A., Jaeger R.G., de Araújo N.S. (2007). Primary leiomyosarcoma of the mandible. A case report. Med Oral Patol Oral Cir Bucal.

[39] Rodini C.O., Corrêa Pontes F.S., Rebelo Pontes H.A., da Silva Santos P.S., Gallottini Magalhães M., Santos Pinto D. (2007). Oral leiomyosarcomas: report of two cases with immunohistochemical profile. Oral Surgery, Oral Med Oral Pathol Oral Radiol Endodontology..

[40] Castaldi A., Arcuri T., Carta M., Quilici P., Derchi L.E. (2006). Primary leiomyosarcoma of the oral tongue: magnetic resonance and ultrasonography findings with histopathologic correlation. Acta Radiol.

[41] Yang S.W., Chen T.M., Tsai C.Y., Lin C.Y. (2006). A peculiar site of leiomyosarcoma: the tongue tip-report of a case. Int J Oral Maxillofac Surg.

[42] Cheng C.-Y., Chang K.-M., Chang K.-W., Chang C.-H., Liu C.-J., Kushner G.M. (2004). Rapidly growing mass in mandibular gingiva. J Oral Maxillofac Surg.

[43] Prasad K.C., Alva T.B., Khadilkar U., Madhu D. (2004). Leiomyosarcoma of the maxillary sinuses: report of two cases. Ear Nose Throat J.

[44] Ikram M., Ahmed I., Ahmed D., Ahmed Y.I. (2003). Leiomyosarcoma of the maxilla with spinal metastasis: a case report. Ear Nose Throat J.

[45] Lo Muzio L., Favia G., Farronato G., Piattelli A., Maiorano E. (2002). Primary gingival leiomyosarcoma. A clinicopathological study of 1 case with prolonged survival. J Clin Periodontol.

[46] Montgomery E., Goldblum J.R., Fisher C. (2002). Leiomyosarcoma of the head and neck: a clinicopathological study. Histopathology.

[47] Wada S., Yue L., Furuta I., Takazakura T. (2002). Leiomyosarcoma in the maxilla: A case report. Int J Oral Maxillofac Surg.

[48] Nikitakis N.G., Lopes M.A., Bailey J.S., Blanchaert R.H., Ord R.A., Sauk J.J. (2002). Oral leiomyosarcoma: Review of the literature and report of two cases with assessment of the prognostic and diagnostic significance of immunohistochemical and molecular markers. Oral Oncol.

[49] Dios P.D., Teijeiro J.C., Anguira F.B., Scully C., García E.V., García-García A. (2001). Synchronous oral leiomyosarcoma and squamous cell carcinoma. Oral Surgery, Oral Med Oral Pathol Oral Radiol Endodontology.

[50] Sumida T., Hamakawa H., Otsuka K., Tanioka H. (2001). Leiomyosarcoma of the maxillary sinus with cervical lymph node metastasis. J Oral Maxillofac Surg.

[51] Dry S.M., Jorgensen J.L., Fletcher C.D. (2000). Leiomyosarcomas of the oral cavity: an unusual topographic subset easily mistaken for nonmesenchymal tumours. Histopathology.

[52] Lo Muzio L., Favia G., Mignogna M.D., Piattelli A., Maiorano E. (2000). Primary intraoral leiomyosarcoma of the tongue: an immunohistochemical study and review of the literature. Oral Oncol.

